# Immature Dengue Virus Is Infectious in Human Immature Dendritic Cells via Interaction with the Receptor Molecule DC-SIGN

**DOI:** 10.1371/journal.pone.0098785

**Published:** 2014-06-02

**Authors:** Mareike K. S. Richter, Júlia M. da Silva Voorham, Silvia Torres Pedraza, Tabitha E. Hoornweg, Denise P. I. van de Pol, Izabela A. Rodenhuis-Zybert, Jan Wilschut, Jolanda M. Smit

**Affiliations:** 1 Department of Medical Microbiology, University of Groningen and University Medical Center Groningen, Groningen, The Netherlands; 2 Grupo Immunovirología, Universidad de Antioquia, Medellín, Colombia; University of Rochester, United States of America

## Abstract

**Background:**

Dengue Virus (DENV) is the most common mosquito-borne viral infection worldwide. Important target cells during DENV infection are macrophages, monocytes, and immature dendritic cells (imDCs). DENV-infected cells are known to secrete a large number of partially immature and fully immature particles alongside mature virions. Fully immature DENV particles are considered non-infectious, but antibodies have been shown to rescue their infectious properties. This suggests that immature DENV particles only contribute to the viral load observed in patients with a heterologous DENV re-infection.

**Methodology/Principal findings:**

In this study, we re-evaluated the infectious properties of fully immature particles in absence and presence of anti-DENV human serum. We show that immature DENV is infectious in cells expressing DC-SIGN. Furthermore, we demonstrate that immature dendritic cells, in contrast to macrophage-like cells, do not support antibody-dependent enhancement of immature DENV.

**Conclusions/Significance:**

Our data shows that immature DENV can infect imDCs through interaction with DC-SIGN, suggesting that immature and partially immature DENV particles may contribute to dengue pathogenesis during primary infection. Furthermore, since antibodies do not further stimulate DENV infectivity on imDCs we propose that macrophages/monocytes rather than imDCs contribute to the increased viral load observed during severe heterotypic DENV re-infections.

## Introduction

Dengue virus (DENV), a flavivirus within the *Flaviviridae* family, is the most common mosquito-borne viral infectious agent worldwide. According to new estimates, 390 million DENV infections occur annually, of which about 100 million are symptomatic [1]. There are four different serotypes of DENV. Each of them can cause disease ranging from rather mild dengue fever to more severe dengue hemorrhagic fever and dengue shock syndrome [2], [3]. Pre-existing heterotypic antibodies represent a major risk factor for the development of severe disease via antibody-dependent enhancement of disease (ADE) [4]–[6]. In ADE, pre-existing cross-reactive antibodies are hypothesized to bind to the newly infecting virus and facilitate efficient replication in Fcy-receptor-expressing cells, thereby increasing the infected cell mass and viral load. A high viral load is often a prelude for severe disease development [7].

DENV-infected cells secrete a heterogeneous population of virions that vary in maturation state [8]–[12]. Junjhon and colleagues showed that more than 90% of the particles secreted from C6/36 cells contain at least some prM molecules [8]. Structural work demonstrated that many DENV particles have mosaic structures with immature “spiky” regions and mature “smooth” regions [13]. Fully immature particles are considered non-infectious, as functional analysis in multiple cell lines indicated that the prM protein affects virus-receptor interaction and membrane fusion activity. Earlier studies revealed that the precursor membrane (prM) protein, the hallmark of immature virus particles, caps the envelope (E) protein [14]–[18]. Indeed, furin-dependent cleavage of prM to M is a prerequisite for membrane fusion and infectivity [16], [18]–[22]. Interestingly, antibodies have been found to rescue the infectivity of fully immature DENV particles by Fcy-receptor-mediated binding and cell entry of DENV-immune complexes. Upon cell entry, the prM protein is cleaved by furin present within the endosome to render the particle infectious [17], [19], [23]. This suggests that fully immature particles are only infectious in presence of antibodies and therefore contribute to the viral load observed in secondary DENV infections.

Immature dendritic cells (imDCs) represent important target cells for DENV replication [24]. Virus-cell binding is facilitated through interaction of the glycan moieties that are linked to the E glycoprotein with the receptor molecule Dendritic Cell-Specific Intercellular adhesion molecule-3-Grabbing Non-integrin (DC-SIGN) [25]. Interestingly, a recent report has shown that partially immature particles of West Nile Virus (WNV) – like DENV a member of the flavivirus genus – can infect cells that are engineered to express DC-SIGN [26]. The glycan moieties on prM were found to interact with DC-SIGN, thereby facilitating virus binding and cell entry. In view of the above studies we here assessed the infectivity of fully immature DENV in DC-SIGN expressing cells including primary human monocyte-derived imDCs in the presence and absence of anti-DENV antibodies and compared it with the infectivity of immature DENV in macrophage-like cells.

## Materials and Methods

### Cell culture

Human peripheral blood mononuclear cells (PBMCs) were isolated by standard density centrifugation using Ficoll-Paque Plus (GE Healthcare, Sweden) from buffy coats obtained with written informed consent from healthy, anonymous volunteers, in line with the declaration of Helsinki (Sanquin Bloodbank, Groningen, the Netherlands). All samples were analyzed anonymously. Monocytes were isolated by gelatin adherence [27] and allowed to differentiate in RPMI (Life Technologies) supplemented with 20% fetal bovine serum (FBS), 500U/ml granulocyte-macrophage colony-stimulating factor (GM-CSF), and 250 U/ml recombinant human interleukin-4 (rIL-4) (both from Prospec-Tany, Israel). The medium was replaced every second day till day 6 to generate imDCs. P338D1 cells (American Tissue Culture Collection [ATCC] # CCL-46), a macrophage-like cell line expressing Fcy-receptors, was maintained in DMEM (PAA Laboratories, Austria) supplemented with 10% FBS, 100 U/ml penicillin, 100 mg/ml streptomycin, 0.75% sodium bicarbonate (Invitrogen) and 1 mM sodium pyruvate (Gibco). Vero-WHO cells (European Collection of Cell Culture # 88020401) were maintained in DMEM supplemented with 5% FBS, 100 U/ml penicillin and 100 mg/ml streptomycin. Human adenocarcinoma LoVo cells (ATCC # CCL-229) were maintained in Ham's medium (Life Technologies) supplemented with 20% FBS. B cell lines Raji wild type (wt, ATCC # CCL-86) and Raji DC-SIGN were maintained in RPMI (Life Technologies) supplemented with 10% FBS, 100 U/ml penicillin and 100 mg/ml streptomycin. The Raji DC-SIGN cell line was stably transfected with a plasmid coding for DC-SIGN [28]. All mammalian cells and cell lines where maintained at 37°C/5% CO_2_. C6/36 (ATCC # CRL-1660), an *Aedes albopictus* cell line, was maintained in minimal essential medium (Life Technologies) supplemented with 10% FBS, 25 mM HEPES, 7.5% sodium bicarbonate, 100 U/ml penicillin, 100 mg/ml streptomycin, 200 mM glutamine and 100 mM nonessential amino acids at 30°C/5% CO_2_.

### Flow cytometry

The phenotype of the cultured primary imDCs was confirmed at day 6 using flow cytometry analysis, essentially as described before [27], [29]. The cells were analyzed using the primary labeled antibodies Lineage1-FITC, CD11c-APC, HLR-DR-V500, CD80-PE, CD83-PECy7 and CD86-V50 (Becton Dickinson). DC-SIGN levels of imDCs, Raji wild type, and Raji DC-SIGN cells were determined using an anti-DC-SIGN antibody and a secondary PE-labeled antibody (both R&D systems, MN, USA). Flow cytometry analysis was performed on a LSR-II (Becton Dickinson). Data was analyzed using Kaluza 1.2.

### Viruses

For the functional experiments, standard (std) DENV-1 (strain 16007), DENV-2 (strain 16681), DENV-3 (strain H87) and DENV-4 (strain 1036), were produced in the *Aedes albopictus* cell line C6/36, essentially as described before [17]. Briefly, an 80% confluent monolayer of cells was infected at multiplicity of infection (MOI) of 0.1. Depending on the serotype, 72 to 168 hours post infection (hpi), virus was harvested. Immature DENV-1, DENV-2, DENV-3 and DENV-4 were produced LoVo cells as described. Briefly, an 80% confluent monolayer of cells was infected at MOI 2 and 72 hpi immature DENV was harvested. LoVo cells are deficient in the protease furin, therefore no cleavage of prM to M can occur and the secreted virus particles are fully immature, which has been characterized by us before [22]. The specific infectivity was determined at 72 hpi following std DENV and immature DENV infection at MOI 2 for all serotypes.

### Infectivity assays

imDCs were infected at a multiplicity of genome-containing particles (MOG) of 1000 of either immature DENV-2 or std DENV-2. At 1.5 hpi, fresh medium was added to the cells. Growth curve analysis showed that DENV-infected imDCs start to secrete new particles at 24 hpi (data not shown). We decided to harvest at 43 hpi so we could measure the maximum output from the first round of replication. The number of produced infectious particles was measured by standard plaque assay on BHK-21 clone 15 cells. The detection limit of the plaque assay is 18 PFU/ml [30]. The role of DC-SIGN was studied by incubating imDCs 1 h before and during infection with 25 µg/ml of either an anti-DC-SIGN antibody or a non-specific isotype control (both R&D systems, MN, USA). To test if viral infectivity could be enhanced by antibodies, immature DENV-2 (MOG 1000) or, as a control, std DENV-2 (MOG 100) was pre-opsonized with 10-fold sequential dilutions of human serum before infection. We used convalescent serum (28 days following infection) from a DENV-2 immune, hospitalized patient. For gain-of-function experiments, early passages of the stably transfected B cell line Raji DC-SIGN and as a control, Raji wt were infected with MOG 1000 of immature or std DENV-1, 2 and 4 under the same conditions as described above. Post-entry maturation of immature particles was blocked by treating cells with the furin inhibitor (FI) Decanoyl-RVKR-CMK (Calbiochem) prior (50 µM) and during (25 µM) virus infection. Infectivity assays on the macrophage-like cell line P388D1 were performed under the same conditions as for imDCs. For antibody-dependent enhancement studies, P388D1 cells were infected with human serum-opsonized immature DENV-1, 2, and 4 at MOG 1000 or, as a control, non-opsonized std DENV-1, 2, and 4 at MOG 1000. For experiments in P388D1 and Raji cells, the number of infectious particles was determined by immunofocus assay, as described below.

### qPCR

The number of genome-containing particles (GCPs) was determined by quantitative PCR (qPCR) according to an earlier published protocol based on DENV-2 [12]. Briefly, the DNA was amplified for 40 cycles of 15 s at 95°C and 60 s at 55°C (DENV-3) or 60°C (other serotypes). Determination of the number of RNA copies was performed with a standard curve (correlation co-efficient >0.995) of quantified DENV plasmids constructed with standard DNA techniques. For DENV-1: pcDNA3 encoding the M protein sequence of DENV-1 strain 16007; DENV-3: pcDNA3 encoding the E protein sequence of DENV-3 strain 16562; and DENV-4: pcDNA3 encoding the E protein sequence of DENV-4 strain 1036 was used. The details of the primers and probes used for DENV-1, 3 and 4 can be found in table S1.

### Immunofocus assay

To assess the specific infectivity of immature and std DENV of the different serotypes, the number of infectious particles was determined by an adapted protocol for immunofocus assay [31]. One day before titration, 1.3×10^4^ Vero-WHO cells were seeded per well in a 96-well plate. Prior to infection, the medium was removed and cells were infected with 10-fold serial dilutions of the virus. After 1.5 h incubation at 37°C, MEM/2% FBS and 1% carboxymethylcellulose (Sigma-Aldrich, Steinheim, Germany) was added as overlay. Cells were fixed and stained after 2 days (DENV-4), 3 days (DENV-1) or 4 days (DENV-2 and DENV-3) of incubation at 37°C/5% CO_2_. Prior to the staining procedure, cells were fixed with 10% formaldehyde in phosphate-buffered saline (PBS). Subsequently, cells were washed with PBS and permeabilized with 2% Triton-X. For detection, 4G2 antibody (Millipore, Temecula, CA) was used as a primary antibody and goat anti-mouse HRP-labeled antibody (Southern Biotech, Birmingham, AL) as a second antibody. Cells were stained with Trueblue Peroxidase Substrate (KPL, Gaithersburg, MD). The foci were counted manually. The limit of detection for immunofocus assay is 20 infectious units (IU) per ml.

### Statistical analysis

Statistical analysis was performed with the GraphPad Prism 5 software. All data were analyzed using the Mann Whitney U-test. P values <0.05 were considered statistically significant. P values are depicted in the figures as * (<0.05), ** (<0.01), and *** (<0.001).

## Results

### Monocyte-derived immature dendritic cells are susceptible to infection by immature DENV

**Figure 1:**
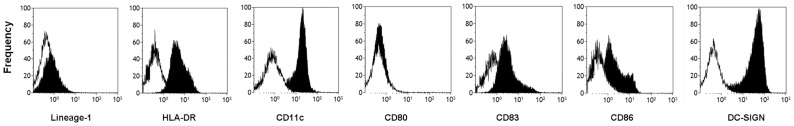
Phenotypic analysis of monocyte-derived immature dendritic cells. Expression profile of different cell type markers by flow cytometry, details are provided in the text. One representative analysis is shown. White curve area: control antibody. Black curve area: specific staining antibody.

Human primary immature dendritic cells (imDCs) were obtained upon culture of PBMC-derived monocytes in the presence of GM-CSF and rIL-4. Six days after culture, the phenotype of the cells was determined by flow cytometry. Figure 1 shows that the cells have a typical imDCs expression pattern: Lin^−^, HLA-DR^+^, CD11c^+^, CD80^−^, CD83^low^ and CD86^low^. Importantly, and as expected, imDCs were found to express high levels of DC-SIGN. The differentiated imDCs were infected at MOG 1000 of either immature DENV-2 or std DENV-2. Immature DENV was produced on furin-deficient LoVo cells. We showed before that LoVo-derived DENV has an average prM content of 94±9%, demonstrating that LoVo-derived DENV is fully immature [11].The prM protein is known to control viral infectivity [11], [14], [18], [20], [21], and we observed before that the specific infectivity of LoVo-derived DENV is at least 10,000 fold reduced compared to that of std DENV generated in C6/36 cells [11], [17], [32]. This is comparable to the drop in specific infectivity of a furin cleavage-deficient recombinant DENV (pDENprMÄ90) [17]. Furthermore, the reduced specific infectivity of LoVo-derived DENV was rescued upon exogenous treatment with furin, indicating that prM to M cleavage is a prerequisite for viral infectivity [11]. Taken together, prM to M cleavage of DENV is solely mediated by furin and LoVo cells can be used to generate fully immature DENV particles. The specific infectivity of the immature DENV-2 batch used in this study was ∼100,000 fold reduced compared to that of std DENV-2, again demonstrating that immature DENV-2 is essentially non-infectious in BHK-21-15 cells. The GCP to PFU ratio was 8.4×10^6^ for immature DENV compared to 73 for std DENV.

**Figure 2:**
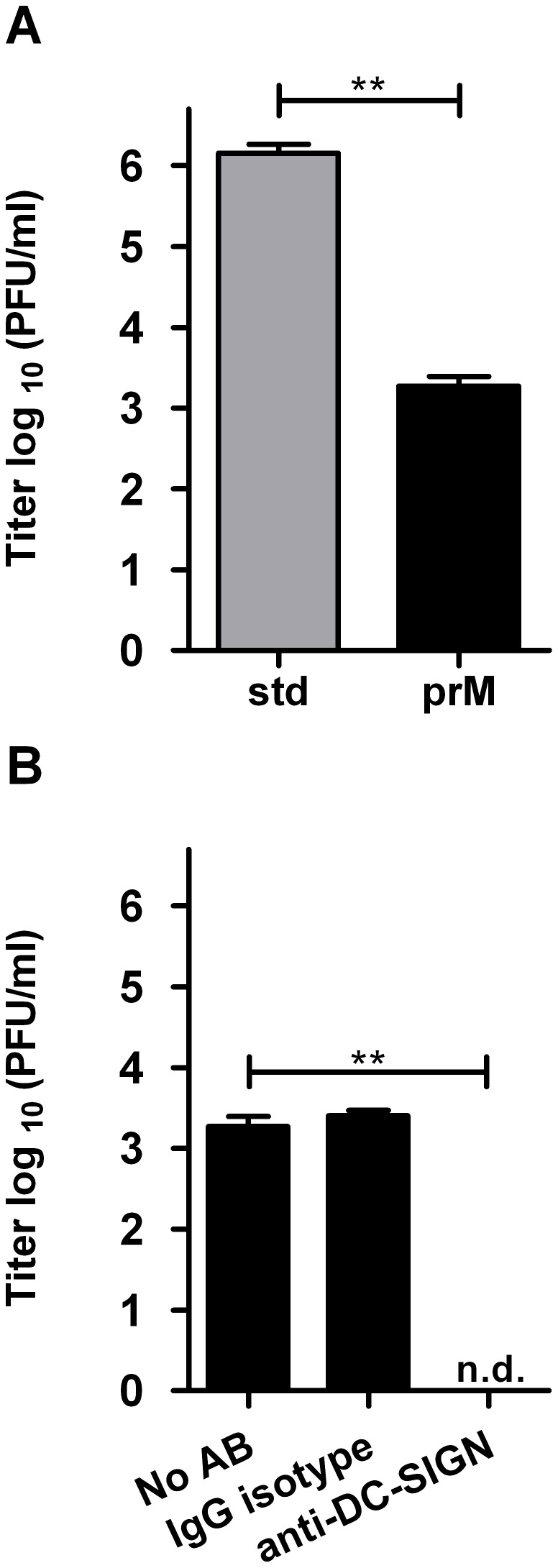
Fully immature DENV-2 particles exhibit basic infectivity on immature dendritic cells. imDCs were infected with MOG 1000 of standard (std) or immature DENV-2. Supernatant was harvested 43 hpi and analyzed. (A) DENV-2 infectivity on imDCs. (B) Role of DC-SIGN on immature DENV-2 infectivity in imDCs as tested by DC-SIGN blockage. Limit of detection is 18 PFU/ml. Data are expressed as means of at least two independent experiments performed in triplicate; error bars represent standard error of the mean (SEM). N.d. denotes for “not detectable”. Levels of significance (Mann Whitney U test) are presented as **  =  p<0.01.

**Figure 3:**
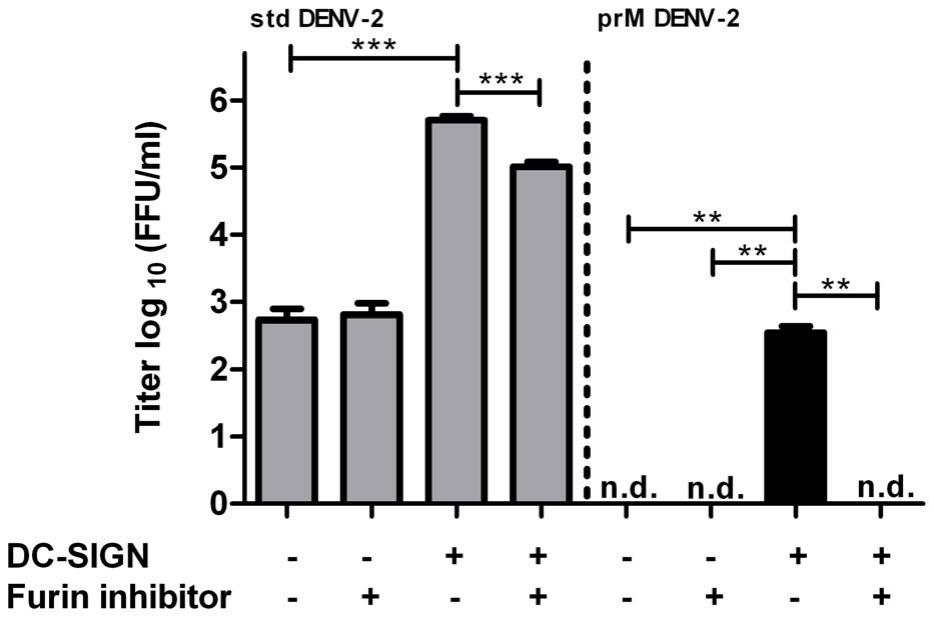
Immature DENV-2 can infect Raji DC-SIGN cells in a furin-dependent manner. Raji DC-SIGN cells and Raji wt cells were infected with MOG 1000 of std (grey bars) or immature (black bars) DENV-2. Prior and during infection, selected samples were treated with furin inhibitor. Supernatant was analyzed for viral progeny at 43 hpi. Limit of detection is 20 FFU/ml. Data are expressed as means of at least two independent experiments performed in triplicate; error bars represent SEM. N.d. denotes for “not detectable”. Levels of significance (Mann Whitney U test) are presented as **  =  p<0.01 and ***  =  p<0.001.

Although we never detected infectivity of immature DENV-2 in monocyte and macrophage cell lines or human PBMCs [17], [32], we did observe low-level infectivity of immature DENV-2 in imDCs (Figure 2A). At 43 hpi, 1.8×10^3^ PFU/ml were produced following infection with immature DENV, while for std virus a titer of 1.4×10^6^ PFU/ml was obtained. Similar results were observed in cells of another blood donor (data not shown). Furthermore, to test whether immature DENV infects imDCs due to interaction with DC-SIGN, we treated imDCs with either an anti-DC-SIGN antibody or a non-specific isotype control. Indeed, blockage of the DC-SIGN receptor completely abrogated infection of imDCs with immature DENV (Figure 2B), indicating that DC-SIGN acts as an entry receptor for immature virions. To further confirm the importance of DC-SIGN in facilitating entry of immature DENV, we next tested the infectious properties of DENV-2 on Raji wt and Raji DC-SIGN expressing cells. Analysis by flow cytometry indicated that 96.4–88.4% of Raji DC-SIGN cells were positive for DC-SIGN, whereas Raji wt cells were negative for DC-SIGN expression. To ensure a high level of DC-SIGN expression, only early passages of cells were used for experiments. As depicted in Figure 3, expression of the receptor molecule DC-SIGN significantly increased viral progeny production following infection with std DENV-2 and immature DENV-2. For immature DENV-2, no virus particle production was detected in cells lacking DC-SIGN expression. We next assessed the importance of furin-mediated cleavage of prM during entry of immature DENV. To this end, cells were treated with furin inhibitor prior and during DENV infection. Treatment of Raji DC-SIGN expressing cells with furin inhibitor caused a small but significant (p<0.001, two-tailed Mann-Whitney test) decrease in virus production following std DENV infection whereas no effect on virus production was seen in Raji wt cells. The observed decrease in infectivity in Raji DC-SIGN cells probably reflects the presence of partially and fully immature virions present within the standard virus preparation that require furin-mediated cleavage upon entry. Importantly, furin inhibitor completely abolished the infectivity of immature DENV-2 on Raji DC-SIGN cells, confirming the importance of prM cleavage for infectivity.

### Infectious properties of DENV-1, DENV-3, DENV-4

After having shown that immature DENV-2 is infectious in imDCs via DC-SIGN interaction, we strived to assess if this observation can be generalized for the other DENV serotypes as well. Since the infectious properties of fully immature DENV-1, 3 and 4 have not been described before, we first examined and compared the specific infectivity of both std and fully immature DENV virions of all four serotypes. For this purpose, we analyzed virus particle production on C6/36 cells and LoVo cells at 72 hpi by RT-PCR [12] and immunofocus assay [31] to measure genome-containing particles and infectious units, respectively. Table 1 shows that immature particles of all four serotypes have a very low specific infectivity on Vero-WHO cells. When compared to the std DENV preparations, the specific infectivity of immature DENV was about 700-fold reduced for DENV-4 and more than 80,000-fold reduced for DENV-2. The specific infectivity of immature DENV-1 and DENV-3 was at least 2000-fold and 300-fold reduced, respectively. The reduction in specific infectivity may be underestimated for DENV-1 and DENV-3 since we were not able to detect any infectivity (limit of detection of the immunofocus assay is 20 IU/ml).

**Table 1:** Specific infectivity of fully immature and standard DENV preparations in epithelial cells.

Serotype	Immature DENV			Standard DENV		
	GCP	FFU	GCP:FFU ratio	GCP	FFU	GCP:FFU ratio
**DENV-1**	7.1×10^7^	N.D.	>3.6×10^6^ *	8.2×10^9^	4.8×10^6^	1698
**DENV-2**	2.7×10^9^	217	1.3×10^7^	2.9×10^7^	1.6×10^5^	182
**DENV-3**	1.7×10^7^	N.D.	>8.5×10^5^ *	2.3×10^8^	1.2×10^5^	1986
**DENV-4**	1.8×10^8^	250	7.3×10^5^	1.2×10^10^	1.1×10^7^	1093

Average results of three independent virus cultures. GCP: Genome containing particles. IU: Infectious units. N.D.: Not detectable. *Based on the detection limit of the immunofocus assay (20 FFU/ml).

**Figure 4:**
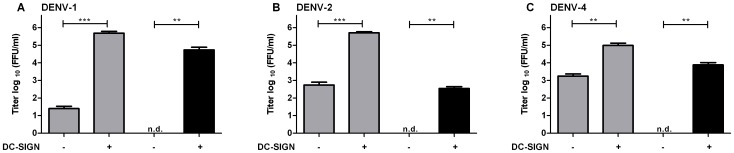
DC-SIGN enhances the permissiveness of Raji cells for both std and immature DENV of different serotypes. Raji DC-SIGN cells and Raji wt cells were infected with MOG 1000 of std (grey bars) or immature (black bars) DENV-1 (A), DENV-2 (B) or DENV-4 (C). Supernatant was analyzed for viral progeny at 43 hpi. Limit of detection is 20 FFU/ml. Data are expressed as means of at least two independent experiments performed in triplicate; error bars represent SEM. N.d. denotes for “not detectable”. Levels of significance (Mann Whitney U test) are presented as **  = p<0.01 and ***  =  p<0.001.

We next attempted to analyze the infectivity of DENV-1, 3, and 4 in imDCs. However, using std virus of these serotypes, only a viral output of about 10^3^ IU/ml was measured at 43 hpi. Unfortunately, the low infectivity of std DENV-1, 3, and 4 in imDCs prevented further characterization of the infectious properties of immature DENV in these cells. To be able to assess the role of DC-SIGN in facilitating immature DENV-1 and 4 entry we decided to use Raji DC-SIGN cells. We did not test immature DENV-3, since it was not possible to propagate this virus to sufficiently high titers (Table 1). Immature DENV-1 and 4 productively infected Raji DC-SIGN cells as 5.4×10^4^ and 7.7×10^3^ FFU/ml were detected respectively. Raji wt cells were not permissive to immature DENV-1 and 4 (Figure 4). Both std DENV-1 and std DENV-4 infected Raji DC-SIGN cells to approximately the same level as std DENV-2 (Figure 4). The effect of DC-SIGN on DENV infectivity was most prominent for DENV-1 suggesting that especially the DENV-1 strain used in our experiments is dependent on DC-SIGN for entry. Taken together, although we could not determine the infectivity of immature DENV other than DENV-2 in imDCs, the above results indicate that DC-SIGN does facilitate infectivity of immature DENV-1, 2, and 4 particles.

### Antibody-mediated enhancement of immature DENV-2

**Figure 5:**
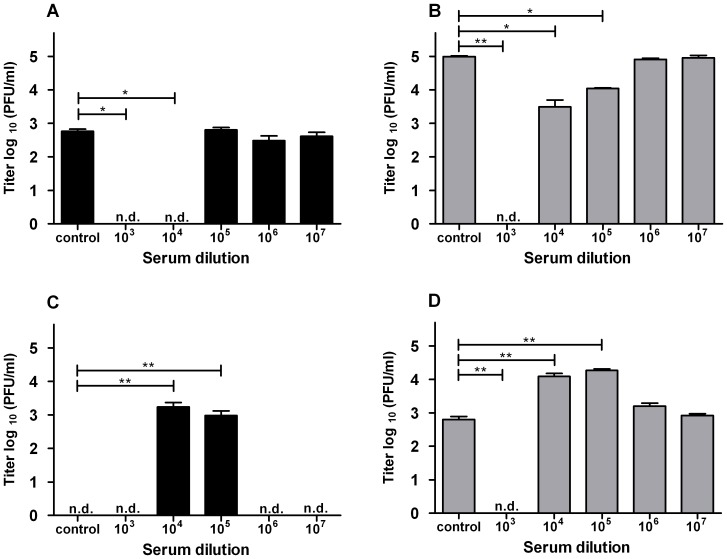
imDCs do not support antibody-dependent enhancement of DENV infection. (A+B) imDCs were infected with immature DENV-2 (A) at MOG1000 and std DENV-2 (B) at MOG 100 as mentioned in the text. Data of one representative donor is shown. For each donor, experiments were performed at least in duplicate. (C+D) P388D1 cells were infected with immature (C) or std (D) DENV-2 at MOG 1000 under similar conditions as in panel A+B. At least two independent experiments were performed in triplicate. Limit of detection is 18 PFU/ml. Error bars represent SEM. N.d. denotes for “not detectable”. Levels of significance (Mann Whitney U test) are presented as *  =  p<0.05, **  =  p<0.01 and ***  =  p<0.001.

Our observation that immature DENV particles are infectious in DC-SIGN expressing imDCs prompted us to also investigate if viral infectivity can be enhanced by anti-DENV serum. Earlier reports performed with std DENV preparations showed that high expression of DC-SIGN is inversely correlated with ADE [29], [33]. However, it is not known if this holds true for infection with immature DENV. Therefore, we performed infectivity assays with immature DENV-2 and, as a control, std DENV-2. Before infection, the virus was pre-opsonized with human serum of an DENV-2 immune individual. As shown in Figure 5A, none of the serum dilutions tested enhanced the basic infectivity of immature DENV in imDCs. Furthermore, and in line with the prior results [29], no enhancement of std DENV infection was observed (Figure 5B). At lower dilutions (≤10^4^ for immature DENV and ≤10^3^ for std DENV), neutralization of viral infectivity was detected. No enhancement of std DENV infectivity was seen upon infection of the cells at lower MOGs (0.1, 1 or 10; data not shown). This shows that the absence of ADE at higher MOG values was not due to saturation of virus particle production capacity in the absence of serum. We achieved comparable results in all donors tested (at least two different donors for each experiment, data not shown), indicating that the outcomes are donor-independent.

To test whether the serum possesses inherent enhancing activity, we next performed infectivity assays in P388D1 cells, a macrophage-like cell line expressing Fcy-receptors. Indeed, and in line with previous published data [17], [32], viral infectivity of immature DENV-2 was enhanced at a serum dilution of 10^4^/10^5^ to std virus levels in the absence of antibodies. We also observed a significant increase in infectivity of std virus at a serum dilution 10^4^/10^5^ (Figure 5D). At low serum dilutions, neutralization of infection was observed (Figures 5C and 5D). In conclusion, the above data fits nicely with the well-described infectivity profile of flaviviruses in presence of antibodies [29], [31], [33], [34].

### Antibody-mediated enhancement of immature DENV-1, 2 and 4 in macrophage-like cells

Under laboratory conditions, enhancement of DENV infectivity can be detected using homotypic antibodies as long as the antibody concentration is lower than the threshold required for neutralization [17], [29], [32], [35]. However, during natural infection, severe disease is predominantly related with heterotypic secondary infection [4], [36]. For this reason, we aimed to assess the influence of heterotypic human serum on the infectivity of different DENV serotypes as well.

**Figure 6:**
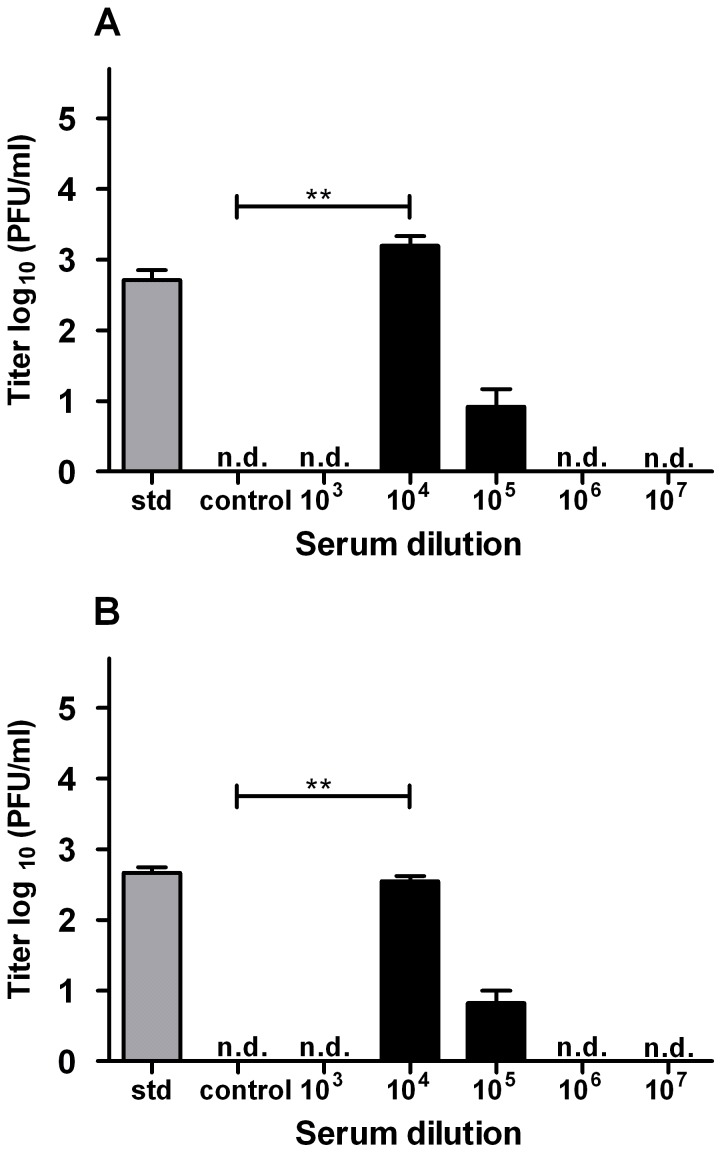
Immature DENV particles of different serotypes can be rendered infectious by heterotypic human serum. P388D1 cells were infected with MOG 1000 of std (grey bars) and pre-opsonized immature (black bars) DENV-1 (A) or DENV-4 (B) as described in the text. At least two independent experiments were performed in triplicate. Limit of detection is 20 FFU/ml. Error bars represent SEM. N.d. denotes for “not detectable”. Levels of significance (Mann Whitney U test) are presented as **  =  p<0.01.

Given the low infectivity of DENV-1, 3 and 4 in imDCs, we evaluated the enhancing properties of immune serum in the macrophage cell line P388D1. In P388D1 cells, the infectivity of std DENV-1 and 4 was comparable to that of DENV-2 (Figure 5D, Figure 6A and 6B). No infectivity was seen for immature DENV-1 and 4 in the absence of serum. We next assessed whether anti-DENV-2 serum stimulated viral infectivity of immature DENV-1 and 4 and revealed that the serum indeed rescues the infectious properties of these viruses. As with DENV-2 (Figure 5), the infectivity increased to levels comparable to std DENV-1 and 4 infection in the absence of antibodies (Figures 5, 6A and 6B). The enhancement profiles were similar for heterotypic and homotypic conditions, which suggests that the human serum used in our study contains a high level of cross-reactive antibodies.

## Discussion

In summary, this study shows that immature DENV particles are infectious in imDCs via interaction with DC-SIGN. The importance of DC-SIGN is underlined by the observation that Raji B cells stably transfected with DC-SIGN are susceptible to immature DENV-1, 2, and 4 infection. Viral infectivity of immature DENV in imDCs is relatively low and cannot be stimulated by antibodies. In contrast, antibodies do enhance infectivity of immature DENV in macrophages.

The E glycoprotein is responsible for efficient interaction of the virus with host cells during primary infection. In immature particles, the E protein is obscured by prM, prohibiting efficient virus-receptor interaction [15], [16], [19]. Therefore, immature particles are presumably scored non-infectious in numerous cell lines like K562, U937, THP-1, P388D1, and human PBMCs [17], [22], [25]. However, and in line with recent results on WNV [26], we here show that immature DENV is infectious in cells expressing DC-SIGN. Binding of immature particles to DC-SIGN is presumably facilitated by sugar groups linked to position Asn^69^ on prM, or sugar groups linked to position Asn^67^ and Asn^153^ on E [15], [16], [26], [28]. In line with earlier studies, we show that DC-SIGN has a more prominent role in DENV-1 infectivity than in DENV-2 and 4 [37], [38]. This result is however strain-dependent as other studies do not show a difference in DC-SIGN dependence between DENV-1, 2, 4 [39], [40]. The more pronounced effect of DC-SIGN on DENV-1 strain 16007 infectivity is also observed for immature virus as the progeny viral titer following immature DENV-1 infection is 1–2 log higher compared to that of DENV-2 and 4.

Upon binding, DENV enters imDCs via an as yet unknown pathway [41]. For partially immature WNV particles, it has been shown that furin cleavage of prM upon cell entry is not strictly required for infection of Raji DC-SIGN cells [26]. Here, and in line with other studies [19], [27], [30], we show that fully immature particles do require furin processing for infectivity. This confirms that prM to M cleavage is a prerequisite for infectivity. It is not fully understood however how the pr peptide is released from the virion after furin cleavage. Kielian and co-workers elegantly showed that the pr-E interaction is tightly controlled by pH [19] and hypothesized that the pr peptide remains associated within the mildly acidic lumen of the secretory pathway but is released in the more acidic environment of endosomes thereby allowing membrane fusion. The number of prM molecules necessary to support furin-dependent or furin-independent infectivity immature flavivirus particles is unknown so far.

The infectious potential of immature DENV in imDCs may imply that these particles contribute - albeit limited - to the total viral load during primary infection. Furthermore, immature particles may be important in the initiation of infection as virus particles produced in mosquito cells are known to have a high prM content [8], [9], [22]. On the other hand, we previously showed that immature WNV particles do not cause disease in mice when injected through the intraperitoneal route [32], [42]. Though, during natural infection, the virus is inoculated in the skin and directly encounters Langerhans cells [43]. These cells express the C-type lectin langerin, but no DC-SIGN [44]. Whether Langerhans cells are permissive to immature DENV infection remains to be elucidated.

Neither std DENV nor immature DENV exhibit ADE on imDCs during heterotypic re-infection. In line with previous observations [33], we propose that Fcy-receptors expressed on imDCs do not have an additive effect on viral infectivity due to the high cell surface expression of DC-SIGN. It is, however, possible that the antibody–opsonized complexes are internalized through the Fcy-receptor without net increase in viral infectivity. Furthermore, the observation that antibody-opsonized immature DENV has a lower infectivity than std DENV may suggest that imDCs are less efficient in promoting virus maturation upon entry than macrophages. Other target cells like monocyte or macrophage-like cell lines do support enhanced infection of antibody-opsonized immature and std DENV via Fcy-receptor-mediated entry, thereby increasing total viral output [17], [29].

Our results suggest that imDCs generate a similar amount of virus progeny during primary and secondary heterotypic infection, but do not contribute to the increase of viral load seen in secondary heterotypic infection.

## Supporting Information

Table S1 **Primer and probes used for cDNA generation and quantitative PCR.** Primers and probes described in the table were used for the determination of genome-containing particles of each serotype.
(DOC)
