# Supplementary material for: Immature Dengue Virus Is Infectious in Human Immature Dendritic Cells via Interaction with the Receptor Molecule DC-SIGN
Source: PLoS One. 2014 Jun 2;9(6):e98785. doi: 10.1371/journal.pone.0098785 (PMC4041791; doi:10.1371/journal.pone.0098785)
Supplement: Table S1 — Primer and probes used for cDNA generation and quantitative PCR. Primers and probes described in the table were used for the determination of genome-containing particles of each serotype. (DOC) [file pone.0098785.s001.doc]

**Table S1**. Primer and probes used for cDNA generation and quantitative PCR

| **Serotype** | **Forward primer** | **Reverse primer** | **TaqMan Probes** |
| --- | --- | --- | --- |
|  |  |  |  |
| DENV-1 | 5’-TGCTCTCAAACTGGCGAACA-3’ | 5’-TCCAAGCACCTTCAGAGGACAT-3’ | 5’-FAM-CCGTCGCATTGGCCCCACA-TAMRA-3’ |
| DENV-3 | 5’-AGGGAGTCACGGCTGAGATAAC-3’ | 5’-CCGAGGGTTCCATATTCAGGTA-‘3 | 5’-FAM-CCCCAGGCATCAACCGCTGAA-TAMRA-3’ |
| DENV-4 | 5’-CATGGGTCGATCTGGTGCTA-3’ | 5’-CCTTGGCTGTTGTCTTAGTCA-3’ | 5’-FAM-CATGGCCCAGGGAAAACCAACCTT-TAMRA-3’ |

Primers and probes described in the table were used for the determination of genome-containing particles of each serotype.
